# High adherence to angiotensin-converting enzyme inhibitor in children and adolescents with Alport syndrome: objective verification using liquid chromatography-mass spectrometry

**DOI:** 10.1007/s00467-025-07053-0

**Published:** 2025-11-22

**Authors:** Jan Boeckhaus, Burkhard Tönshoff, Lutz T. Weber, Dieter Haffner, Lars Pape, Kay Latta, Henry Fehrenbach, Baerbel Lange-Sperandio, Matthias Kettwig, Sabine König, Ulrike John-Kroegel, Jutta Gellermann, Matthias Galiano, Angelika Hafke, Frank Streit, Oliver Gross

**Affiliations:** 1 Clinic for Nephrology and Rheumatology, University Medical Center Göttingen, Goettingen, Germany; 2 Department of Pediatrics I, University Children’s Hospital Heidelberg, Heidelberg, Germany; 3 Pediatric Nephrology, Children’s and Adolescents’ Hospital, University of Cologne, Faculty of Medicine and University Hospital Cologne, Cologne, Germany; 4 Department of Pediatric Kidney, Liver and Metabolic Diseases, Hannover Medical School, Hannover, Germany; 5 Department of Pediatrics II, University Children’s Hospital, University of Duisburg-Essen, Essen, Germany; 6 Clementine Kinderhospital Frankfurt, Frankfurt, Germany; 7 Pediatric Nephrology, Children’s Hospital, Memmingen, Germany; 8 Dr. V. Hauner Children’s Hospital, Ludwig Maximilians University, Munich, Germany; 9 Department of Pediatrics and Adolescent Medicine, University Medical Center Göttingen, Göttingen, Germany; 10 University Children’s Hospital Münster, Münster, Germany; 11 Division of Pediatric Nephrology, University Children’s Hospital, Jena, Germany; 12 Pediatric Nephrology, Charité Children’s Hospital, Berlin, Germany; 13 Department of Pediatrics and Adolescent Medicine, University Hospital, Friedrich-Alexander-University Erlangen, Erlangen, Germany; 14 Department of Clinical Chemistry, University Medical Center Göttingen, Göttingen, Germany

**Keywords:** Hereditary kidney disease, Alport syndrome, Adherence, Clinical trial, Ramipril

## Abstract

**Background:**

Kidney failure (KF) in children and adolescents leads to reduced lifespan and compromised health. Alport syndrome (AS) is a leading hereditary cause of KF in children. Angiotensin-converting enzyme inhibitors (ACEi) have demonstrated efficacy in delaying KF in young people living with AS, but non-adherence can compromise their therapeutic benefits. To investigate the adherence to ACEi in children and adolescents with AS, a liquid chromatography-mass spectrometry (LCMS)-based method was developed for objective verification of recent medication intake at two different time points in this cohort study.

**Methods:**

Urine samples from 58 children enrolled in the EARLY PRO-TECT Alport trial were analyzed. An LCMS-based method was established and validated to simultaneously screen and quantify both ramipril and ramiprilat in urine samples. Participants were not informed in advance of the medication intake measurements.

**Results:**

A total of 106 urine samples from 58 patients with early stages of chronic kidney disease (mean estimated glomerular filtration rate, 130 ± 32 mL/min/1.73 m^2^) were analyzed at two different time points. All 13 negative control samples (100%; 95% confidence intervals [CI] 75.7% to 100%) were identified correctly. Adherence to ACEi at the time of sampling was consistently high, with 96% (47/49; 95% CI 86% to 99.5%) and 95% (42/44; 95% CI 84.5% to 99.4%) of children showing confirmed drug intake at initial and second adherence measurements.

**Conclusion:**

This study demonstrated that children with chronic kidney disease, when treated with ACEi within a clinical trial, show high adherence to the prescribed medication.

**Graphical abstract:**

**Figure:**
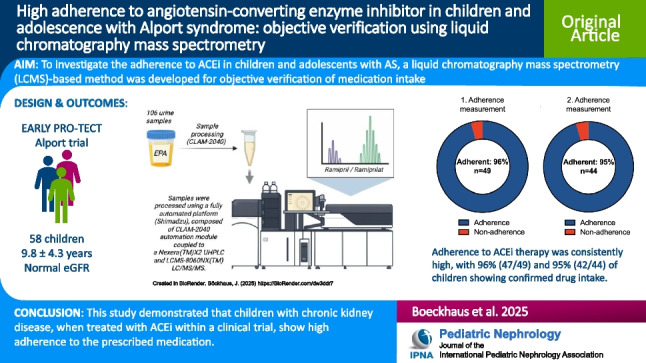
A higher resolution version of the Graphical abstract is available as Supplementary information

**Supplementary Information:**

The online version contains supplementary material available at 10.1007/s00467-025-07053-0.

## Background

Kidney failure (KF) leads to reduced lifespan in children and adolescents [1, 2]. Alport syndrome (AS) is a leading hereditary cause of KF in children [3–5]. In most registries, CAKUT is the most common pediatric cause of kidney failure, and the relative frequency of Alport syndrome varies by region. AS is caused by a defect in type IV collagen, which is an essential component of the basement membrane in the kidney, eyes, and cochlea [6, 7]. The disruption of type IV collagen structure results in impaired function of the glomerular basement membrane filtration barrier, leading to hematuria, increased albuminuria, and, consequently, a high risk for progression to KF [8]. The disease often manifests further with progressive sensorineural hearing loss and ocular lesions [9–11]. The predominant mode of inheritance for AS is X-linked, attributed to pathogenic variants within the *COL4A5* gene. Autosomal recessive or dominant inheritance patterns, affecting the *COL4A3* or *COL4A4* genes, account for up to 30% [12–15]. Furthermore, digenic inheritance has been reported [16, 17]. Renin-angiotensin system inhibition (RASi) with angiotensin-converting enzyme inhibitors (ACEi) or angiotensin receptor blockers (ARBs) has demonstrated efficacy in delaying KF in patients with AS [18–20]. Ramipril, an ACEi, was investigated in the randomized placebo-controlled EARLY PRO-TECT Alport trial (NCT01485978) and showed a trend towards slowed disease progression with early treatment, without safety concerns compared to placebo [21]. Despite the established benefits of RASi, achieving optimal therapeutic outcomes hinges on patient adherence to prescribed medication [22]. Medication non-adherence has a negative impact on outcomes for children with chronic illnesses, including those with chronic kidney disease (CKD) or KF [23–26]. Due to the long-term requirement for medication intake, the treatment of asymptomatic disease in early CKD, and the often complex treatment regimens in advanced CKD, adherence in pediatric CKD is particularly challenging [27]. Consequently, non-adherence can be a significant obstacle to effective treatment in this vulnerable population, potentially compromising the therapeutic benefits of prescribed treatments [28]. Recognizing the limitations of adherence measures such as self-reporting, a liquid chromatography-mass spectrometry-based method was used to provide objective verification of medication intake [29]. Considering the positive family history often present in patients with AS, and the demonstrated safety of ramipril, we hypothesized that the medication intake at two time points representing adherence to medication in this population would surpass rates previously reported in the literature [21, 30]. To investigate this, we analyzed urine samples collected from children enrolled in the EARLY PRO-TECT Alport trial.

## Methods

### Clinical data and sampling of urine

The participant cohort was previously described as part of the EARLY PRO-TECT Alport trial, a randomized, placebo-controlled, double-blind study with an open-label control arm (ClinicalTrials.gov: NCT01485978) [21, 30–34]. Children aged 2 to 18 years with confirmed AS and normal glomerular filtration rates were enrolled. The treatment period lasted 3 to 6 years. Disease progression was defined as doubling or tripling of albuminuria [21]. The study was conducted in accordance with the Declaration of Helsinki (1964, and its later amendments) and approved by the ethics committees of the University Medical Center Göttingen (AZ 11/06/11) and all participating centers. Written informed consent, including the use of collected biosamples for research purposes, was obtained from all legal guardians, and assent from participants aged 6 and older. From the initial cohort of 66 children in the EARLY PRO-TECT Alport trial, eight participants were not included in this study due to withdrawal of consent (*n* = 3), unavailability of urine samples (*n* = 3), protocol violation (*n* = 1), or being asymptomatic (*n* = 1). For this study, urine samples were selected at two time points: V1, 1.5 years (± 0.5 years) and, if available, V2, 3 years (± 0.5 years) after inclusion in the trial to assess medication adherence.

### Intervention and outcome measures

Stages of AS were defined as stage 0, albuminuria < 30 mg/g creatinine; stage I, albuminuria 30–300 mg albumin/g creatinine; and stage II, albuminuria > 300 mg/g creatinine [35]. The baseline for this study was defined as the first adherence measurement. Participants were not informed in advance of the adherence measurements. Analysis also included samples from the placebo arm (negative controls). The investigator responsible for the analysis of the samples was not informed a priori of the inclusion of negative controls. In this study, adherence was defined as objective evidence of recent medication intake at the time of each study visit, confirmed by the presence of ramipril/ramiprilat in the urine sample. The lower limit of quantification (LLOQ) was 1 µg/L for ramipril and 10 µg/L for ramiprilat [36, 37]. Therefore, samples where the sum of ramipril and ramiprilat was less than 10 µg/L were defined as non-adherent. Additionally, in samples with concentrations above the LLOQ, the blinded investigator responsible for conducting the sample measurements assessed samples as adherent or non-adherent in patients with two measurements, based on baseline values and the trend of the ramipril to ramiprilat ratio. In a second step, to normalize for urine dilution effects, concentrations of ramipril and ramiprilat were normalized to urinary creatinine concentration. The creatinine-normalized LLOQ was calculated using the mean creatinine concentration of this sample cohort (1.8 µg/g creatinine for ramipril and 18 µg/g creatinine for ramiprilat).

### Quantitative analysis of ramipril and ramiprilat

**Fig. 1:**
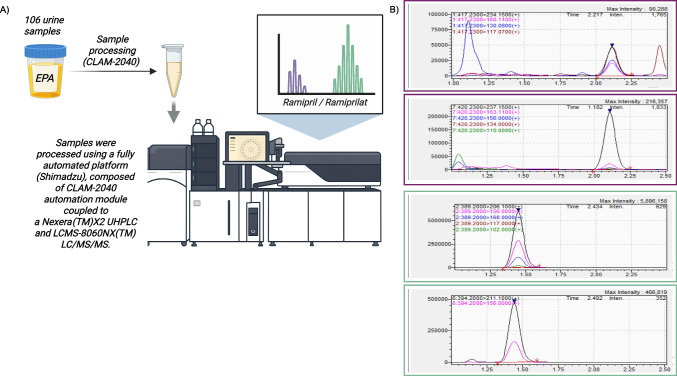
Quantitative analysis of ramipril and ramiprilat in urine using automated liquid chromatography-mass spectrometry. **A** Schematic representation of the analytical workflow (Created in BioRender. Böckhaus, J. (2025) https://BioRender.com/dw3ddr7); **B** representative chromatogram of a urine sample showing low ramipril and high ramiprilat concentrations

Ramipril and ramiprilat in urine were quantified using an automated liquid chromatography-mass spectrometry (LCMS) method (Fig. 1). Calibration standards and quality control samples were prepared using commercially sourced standards. Samples were automatically processed using a CLAM-2040 system (Shimadzu Corporation, Duisburg, Germany) coupled to a Nexera LCMS-8060NX (Shimadzu Corporation, Duisburg, Germany), including protein precipitation and filtration. The chromatographic system consisted of two Shimadzu LC-30AD pumps (NexeraX2), a CTO40AC oven and a SIL-40AC autosampler (Shimadzu Corporation, Duisburg, Germany). Chromatographic separation was achieved using a C18 column with a gradient mobile phase of water and methanol containing acetic acid and ammonium acetate. Mass spectrometric detection was performed using a LCMS-8050 triple quadrupole mass spectrometer (Shimadzu Corporation, Duisburg, Germany) with positive electrospray ionization. Scheduled multiple reaction monitoring (MRM) was utilized for quantification. The method demonstrated linearity across the calibration range and acceptable within- and between-run imprecision.

Lower limits of quantification were established for both ramipril and ramiprilat. Calibration stability was proven over a period of 8 days. A detailed description of the reagents, sample preparation, and LCMS parameters is provided in the supplementary material.

### Statistical methods

Statistical comparisons were not formally powered or prespecified. Continuous variables were presented as mean and standard deviation (SD) or as median and interquartile range (IQR), categorical variables as percentages. For roughly normally distributed values, group means were compared using an unpaired Student’s t-test. Binomial 95% confidence intervals (CIs) were calculated using the Clopper-Pearson exact method. Per-patient concordance metrics were determined using a 2 × 2 contingency table for patients with paired samples. Data analysis was performed using IBM SPSS Statistics (version 30 for MacOS, IBM Corporation, Armonk, NY, USA). GraphPad Prism (version 10 for macOS, GraphPad Software, San Diego, California, USA) and Biorender (Toronto, Ontario, Canada) were used to generate figures.

## Results

### Demographics and clinical characteristics

**Fig. 2:**
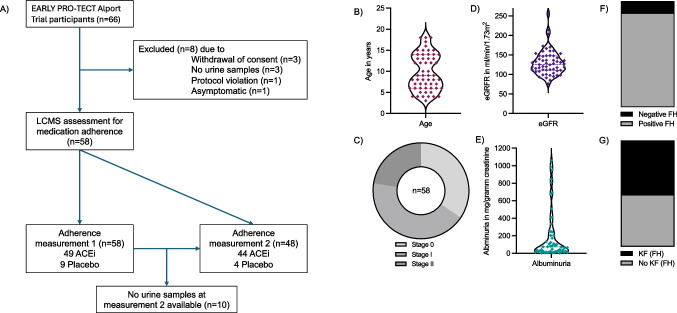
Sampling schema and clinical and demographic characteristics. **A** Sampling schema. ACEi, angiotensin-converting enzyme inhibitor; LCMS, liquid chromatography-mass spectrometry. **B** Age in years (*n* = 58); **C** stages of Alport syndrome (AS) at baseline (n=58); **D** estimated glomerular filtration (eGFR) in mL/min/1.73 m^2^ (*n* = 58); **E** albuminuria in mg/gram creatinine (*n* = 57, two extreme values were not displayed; 2544; 4603 mg/g creatinine); **F** positive and negative family history (FH) for AS (*n* = 58); **G** positive and negative family history for kidney failure (KF) (*n* = 58)

Figure 2 presents a sampling schema and clinical and demographic characteristics of the 58 children included in this study. Of these, 57 were male (98%). The mean age at baseline was 9.8 ± 4.3 years. The mode of inheritance was X-linked in 47 children (81%), autosomal recessive in 9 children (16%), and unknown in 2 children (3%). Twenty children were in AS stage 0 (34%), 25 in AS stage I (43%), and 13 in AS stage II (22%). The mean estimated glomerular filtration rate (eGFR) was 130 ± 32 mL/min/1.73 m^2^, with a median albuminuria of 73 mg/gram creatinine (interquartile range [IQR] 20–250; *n* = 57). At first adherence measurement, 49 children were treated with RASi (84%), with 39 participants in the open-label control arm having already received RASi treatment before inclusion in the trial. The mean duration of RASi treatment at first adherence measurement was 36 ± 29 months, with a maximum duration of 121 months. A positive family history was reported in 88% of the children, with 48% having a family member with KF (Table 1).

**Table 1:** Demographic and clinical characteristics at measurement of adherence

	Number	
Male no. (%)	58	57 (98)
Age (years)	58	9.8 ± 4.3
Mode of inheritance (%)	58	
X-linked		47 (81)
Autosomal recessive		9 (16)
Unknown		2 (3)
AS stage (%)	58	
0		20 (34)
I		25 (43)
II		13 (22)
eGFR	58	130 ± 32 (mL/min/1.73 m^2^)
Albuminuria	57	73 (20–250) (mg/g creatinine)
RASi	58	49 (84)

*RASi* inhibitors of the renin-angiotensin system. *AS stage 0*, albuminuria < 30 mg/g creatinine, *AS stage I* albuminuria 30–300 mg/g creatinine, *AS stage II*, albuminuria > 300 mg/g creatinine. Values are mean + SD, median (IQR), or *n* (%) as appropriate

### Adherence measurement

A total of 106 urine samples were analyzed at two different time points. In urine samples where concentrations were above the LLOQ, the mean ramipril concentration was 46.1 ± 63.1 µg/L (*n* = 70), and the mean ramiprilat concentration was 609.9 ± 623.6 µg/L (*n* = 91) (Table 2; Supplementary Table 1).

**Table 2:** Urine ramipril and ramiprilat concentrations

Analyte	Overall	First adherence measurement (V1)	Second adherence measurement (V2)
Ramipril (µg/L)	46.1 ± 63.1 (70)	46.7 ± 53.6 (36)	45.5 ± 72.6 (34)
Ramiprilat (µg/L)	609.9 ± 623.6 (91)	678.3 ± 686.7 (48)	533.6 ± 542.5 (43)
Sum (ramipril + ramiprilat) (µg/L)	645.5 ± 648.7 (91)	713.5 ± 709.7 (48)	569.7 ± 571.9 (43)

The sum of ramipril and its active metabolite, ramiprilat, is used to provide a comprehensive measure of medication intake. Ramipril, an inactive prodrug, is converted into its active form, ramiprilat. By combining the levels of both compounds, the measurement accounts for individual metabolic differences, offering a more robust indicator of medication adherence. Concentrations below the LLOQ were excluded from this analysis. Numbers in parentheses represent the number of samples above the LLOQ used in the analysis

**Fig. 3:**
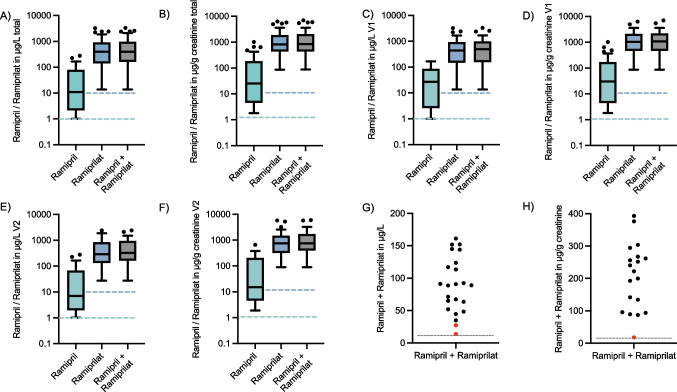
**A**–**F** Tukey plots showing the distribution of all samples with drug levels above the lower limit of quantification (LLOQ). Distribution of all samples in **A** µg/L (*n* = 91) and **B** µg/g creatinine (*n* = 84). Distribution of samples from visit 1 in **C** µg/L (*n* = 48) and **D** µg/g creatinine (*n* = 44). Distribution of samples from visit 2 in **E** µg/L (*n* = 43) and **F** µg/g creatinine (*n* = 40). Dotted lines indicate the LLOQ for ramipril (green) and ramiprilat (blue). Scatter plots of samples with lower ramipril and ramiprilat concentrations in **G** µg/L (*n* = 23) and **H** µg/g creatinine (*n* = 19), data points shown in red were classified as non-adherent

At first adherence measurement, nine children (16%) were not treated with ACEi (negative control). All nine negative control samples (100%) were correctly identified. Of the 49 children receiving ACEi, adherence measurement confirmed recent medication intake in 47 (96%; 95% CI, 86% to 99.5%), while two samples were classified as non-adherent: one due to both ramipril and ramiprilat concentrations being below the LLOQ, and the other because the sum of ramipril and ramiprilat was only marginally above the LLOQ (Fig. 3). These urine samples were from two brothers with X-linked AS in the open-label study arm: a 14-year-old boy (AS stage I) on RASi for 8 years who progressed 1 year after the adherence measurement, and a 13-year-old boy (AS stage II) on treatment with RASi for 10 years who progressed 6 months after entering the EARLY PRO-TECT Alport trial.

At first measurement, adherence was observed in 18 of 20 participants (90%; 95% CI 68.3% to 98.8%) with disease progression and in 29 of 29 participants (100%; 95% CI 88.1% to 100%) without disease progression during the trial. Forty of 42 (95%; 95% CI 84% to 99.4%) with a positive family history demonstrated adherence, compared to 7 of 7 (100%; 95% CI 59.2% to 100%) with a negative family history. Specifically, adherence was confirmed in 21 of 23 participants (91%; 95% CI 72% to 98.9%) with family members affected by KF and in 26 of 26 participants (100%; 95% CI 86.5% to 100%) without family members affected by KF. Furthermore, 37 of 39 pretreated participants (95%; 95% CI 82.9% to 99.4%) showed adherence, while 10 of 10 participants (100%; 95% CI 69.1% to 100%) who started ACEi at inclusion into the clinical trial adhered.

**Fig. 4:**
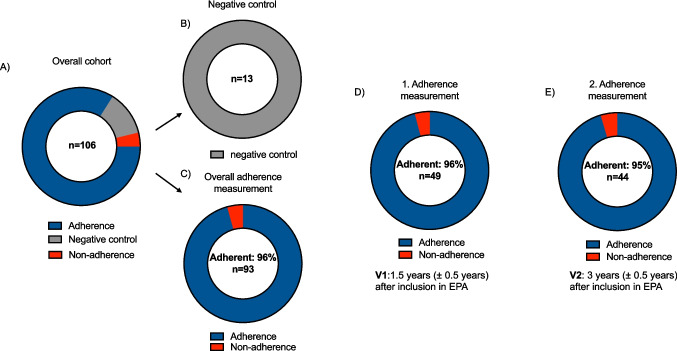
Illustration of medication adherence. Donut graphs show the proportion of **A** the overall cohort of 106 measurements, including 13 negative controls; **B** correctly identified negative controls (*n* = 13, 100%); **C** adherence and non-adherence at both time points of the study (*n* = 93; 96% adherence); **D** adherence and non-adherence at first adherence measurement (*n* = 49; 96% adherence); **E** adherence and non-adherence at the second adherence measurement (*n* = 44; 95% adherence). V1, visit 1; V2, visit 2; EPA, EARLY PRO-TECT Alport trial

In those with available urine samples, a second adherence assessment 1 to 2 years after the first measurement was carried out (*n* = 48). At the second adherence measurement, 44 of the 48 children were receiving ACEi (92%). All four negative controls were correctly identified. Adherence measurement was positive in 42 of 44 children (95%; 95% CI 84.5% to 99.4%) (Fig. 4). Of the non-adherent samples, one exhibited ramipril and ramiprilat concentrations below the LLOQ. The other was classified as non-adherent because concentrations of both analytes were significantly lower compared to the initial adherence measurement. These negative adherence measurements were observed in two boys in AS stage 0 (ages 4 and 6) in the open-label arm. Both had a positive family history, including a family member with KF. Notably, in both children, the initial adherence measurement was positive, and both exhibited no disease progression during the study. In three children, who were switched from placebo (first measurement, negative controls) to ramipril due to disease progression, the second measurement confirmed the intake of ramipril in all three children. In patients with urine measurements above the LLOQ at both time points, the aggregation of ramipril and ramiprilat did not differ significantly (732.7 ± 622.2 µg/L at the first measurement and 583.6 ± 584.4 µg/L at the second measurement; *n* = 40; *p* = 0.27). Per-patient adherence concordance was assessed among 41 children with urine measurements at both time points. The overall percent agreement between the initial and second adherence measurement was 95.1% (39/41 patients). Specifically, 39 of 41 (95.1%) patients were consistently classified as adherent, and zero (0/41) patients were consistently classified as non-adherent.

To investigate the robustness of the analysis against potential urine dilution effects, concentrations of ramipril and ramiprilat were normalized to urinary creatinine concentration using the mean urinary creatinine concentration of this cohort (55.6 mg/dL; *n* = 98). In urine samples where concentrations were above the LLOQ, the mean ramipril concentration was 123 ± 195.4 µg/g creatinine (*n* = 61), and the mean ramiprilat concentration was 1493.2 ± 2140.4 µg/g creatinine (*n* = 84). The classification of all samples remained consistent with the initial analysis using micrograms per liter. The three samples available from patients classified as non-adherent using micrograms per liter were again classified as non-adherent, as two of the samples were below the LLOQ and the second measurement in the third patient was significantly lower compared to the first measurement.

## Discussion

This study examined adherence to the ACEi ramipril in children included in the double-blinded placebo-controlled EARLY PRO-TECT Alport trial at two time points. Using an automated liquid chromatography-mass spectrometry as an objective tool for the determination of ramipril in urine, an adherence rate of 96% and 95% was demonstrated at two distinct time points of the trial. In the literature, self-reported adherence in children with CKD ranges from 74% to 96% [28, 38, 39] Objective measures of medication adherence typically reveal lower rates compared to self-reported adherence, with discrepancies reaching up to 69% [40–42].

The high adherence rates observed in our study may be attributed to several factors: Firstly, the simplified dosing regimen, involving once-daily medication intake, likely facilitated participants’ adherence. Secondly, the frequent presence of a positive family history, providing participants with firsthand insight into the disease, may have enhanced their motivation to adhere to the prescribed therapy. Thirdly, the comprehensive education provided about the therapy’s benefits and potential risks within the clinical trial setting likely enhanced participants’ and their families’ understanding of the study medication [28, 43].

In a clinical setting, the LCMS method can provide objective verification of medication intake, serving as a valuable complement to standard clinical monitoring, such as the measurement of albuminuria. While more expensive, the test’s cost can be justified in specific clinical scenarios where a patient’s adherence is unclear and precise information is needed to guide management.

Furthermore, this study might offer additional insights into ACEi therapy in children with CKD. Notably, despite ACEi therapy at the maximum tolerated dose (up to 6 mg/m^2^), no significant blood pressure reduction was observed in the EARLY PRO-TECT Alport trial [21]. Given this study’s high confirmation of medication intake, non-adherence is an unlikely explanation for this lack of blood pressure reduction. This observation is consistent with prior findings suggesting that an increase in RAS blockade has not consistently been shown to lead to a further drop in blood pressure, which indicates that the nephroprotective effects of ACEi are, at least partially, mediated by mechanisms independent of blood pressure lowering [44–46]. In addition, the high medication intake rate observed in this study, along with the EARLY PRO-TECT Alport trial’s established favorable safety profile of ACEi in pediatric CKD, might be interpreted as an indicator of the therapy’s good tolerability [21].

Strengths of this study include the implementation of adherence measurements after the EARLY PRO-TECT Alport trial, thereby ruling out adherence measurement bias by ensuring participants were not prospectively aware of upcoming controls. The blinding of the scientist who analyzed the samples enhanced the objectivity of the measurements. Furthermore, the reliability of the measurement method was demonstrated by the correct identification of all 13 negative controls.

Additionally, the use of data from a randomized controlled trial enables the description of a clinically well-characterized cohort (including family history and well-monitored disease progression). The use of creatinine-normalized concentrations as a sensitivity analysis indicated that potential urine dilution effects did not confound the classification of patient adherence, as all samples retained their original classification.

As a limitation of this study, samples were not available from all children in the EARLY PRO-TECT Alport trial, resulting in a further reduction of the already limited cohort size. Additionally, the high adherence to medication in this study made it impossible to analyze specific risk factors for non-adherence. Furthermore, the study analyzed samples from primarily boys, limiting the generalizability of the findings to female participants. A further study limitation is the lack of information on participants’ ramipril dosing times (morning or evening), which could have influenced ramipril and ramiprilat concentrations and their interpretation. As a result, a detailed analysis correlating specific dose timing with drug concentration was not feasible. Crucially, this study’s adherence definition prioritizes the detection of ramipril and ramiprilat in urine for regular medication intake, aligning with common adherence definitions in the literature, rather than specific concentration levels [47–49]. Hence, it must be acknowledged that the presence of ramipril or ramiprilat in a single urine sample provides evidence of recent medication intake at a specific moment rather than a continuous measure of ongoing medication intake. In addition, it is important to point out that this study was conducted in a controlled clinical trial setting. This structured environment, with its frequent patient contact and close monitoring, likely contributed to higher adherence rates than those seen in routine practice. Consequently, the generalizability of our findings to routine clinical practice is limited.

Another limitation of this study is the absence of comparator measures (e.g., serum levels) to validate this study’s adherence findings. Future studies should explore these comparisons to better determine the sensitivity and specificity of urine-based methods for measuring adherence, especially in pediatric populations. Overall, this study demonstrated that children with CKD, included in a double-blinded, placebo-controlled clinical trial, show high adherence to the study medication. This provides a compelling rationale for the inclusion of children and adolescents with CKD in clinical trials, a population that could derive substantial benefit from innovative, effective therapies, but is often excluded from clinical trials [50].

## Conclusion

This study demonstrated that children with chronic kidney disease, when treated with ACEi within a clinical trial, show high adherence to the prescribed medication.

## Supplementary Information

Below is the link to the electronic supplementary material.

Supplementary file 1 (DOCX 35.2 KB)

Graphical Abstract (PPTX 1.49 MB)

## Data Availability

Data supporting reported results can be requested from the corresponding author on reasonable request.

## References

[1] Goldstein SL, Graham N, Burwinkle T, Warady B, Farrah R, Varni JW (2006) Health-related quality of life in pediatric patients with ESRD. Pediatr Nephrol 21:846–850. 10.1007/s00467-006-0081-y 16703376 10.1007/s00467-006-0081-y

[2] Chesnaye NC, Schaefer F, Groothoff JW, Bonthuis M, Reusz G, Heaf JG, Lewis M, Maurer E, Paripović D, Zagozdzon I, van Stralen KJ, Jager KJ (2016) Mortality risk in European children with end-stage renal disease on dialysis. Kidney Int 89:1355–1362. 10.1016/j.kint.2016.02.016 27165828 10.1016/j.kint.2016.02.016

[3] Groopman EE, Marasa M, Cameron-Christie S, Petrovski S, Aggarwal VS, Milo-Rasouly H, Li Y, Zhang J, Nestor J, Krithivasan P, Lam WY, Mitrotti A, Piva S, Kil BH, Chatterjee D, Reingold R, Bradbury D, DiVecchia M, Snyder H, Mu X, Mehl K, Balderes O, Fasel DA, Weng C, Radhakrishnan J, Canetta P, Appel GB, Bomback AS, Ahn W, Uy NS, Alam S, Cohen DJ, Crew RJ, Dube GK, Rao MK, Kamalakaran S, Copeland B, Ren Z, Bridgers J, Malone CD, Mebane CM, Dagaonkar N, Fellström BC, Haefliger C, Mohan S, Sanna-Cherchi S, Kiryluk K, Fleckner J, March R, Platt A, Goldstein DB, Gharavi AG (2019) Diagnostic utility of exome sequencing for kidney disease. N Engl J Med 380:142–151. 10.1056/NEJMoa1806891 30586318 10.1056/NEJMoa1806891 PMC6510541

[4] Barker DF, Pruchno CJ, Jiang X, Atkin CL, Stone EM, Denison JC, Fain PR, Gregory MC (1996) A mutation causing Alport syndrome with tardive hearing loss is common in the western United States. Am J Hum Genet 58:1157–1165 8651292 PMC1915056

[5] Puapatanakul P, Miner JH (2024) Alport syndrome and Alport kidney diseases – elucidating the disease spectrum. Curr Opin Nephrol Hypertens 33:283. 10.1097/MNH.0000000000000983 38477333 10.1097/MNH.0000000000000983 PMC10990029

[6] Hudson BG, Tryggvason K, Sundaramoorthy M, Neilson EG (2003) Alport’s syndrome, Goodpasture’s syndrome, and type IV collagen. N Engl J Med 348:2543–2556. 10.1056/NEJMra022296 12815141 10.1056/NEJMra022296

[7] Hudson BG (2004) The molecular basis of Goodpasture and Alport syndromes: beacons for the discovery of the collagen IV family. J Am Soc Nephrol 15:2514–2527. 10.1097/01.ASN.0000141462.00630.76 15466256 10.1097/01.ASN.0000141462.00630.76

[8] Kashtan CE (2000) Alport syndromes: phenotypic heterogeneity of progressive hereditary nephritis. Pediatr Nephrol 14:502–512. 10.1007/s004670050804 10872195 10.1007/s004670050804

[9] Hess K, Pfau M, Wintergerst MWM, Loeffler KU, Holz FG, Herrmann P (2020) Phenotypic spectrum of the foveal configuration and foveal avascular zone in patients with Alport syndrome. Invest Ophthalmol Vis Sci 61:5. 10.1167/iovs.61.2.5 32031577 10.1167/iovs.61.2.5 PMC7324255

[10] Zhang X, Zhang Y, Zhang Y, Gu H, Chen Z, Ren L, Lu X, Chen L, Wang F, Liu Y, Ding J (2018) X-linked Alport syndrome: pathogenic variant features and further auditory genotype-phenotype correlations in males. Orphanet J Rare Dis 13:229. 10.1186/s13023-018-0974-4 30577881 10.1186/s13023-018-0974-4 PMC6303895

[11] Savige J, Sheth S, Leys A, Nicholson A, Mack HG, Colville D (2015) Ocular features in Alport syndrome: pathogenesis and clinical significance. Clin J Am Soc Nephrol 10:703–709. 10.2215/CJN.10581014 25649157 10.2215/CJN.10581014 PMC4386265

[12] Savige J (2018) Should we diagnose autosomal dominant Alport syndrome when there is a pathogenic heterozygous COL4A3 or COL4A4 variant? Kidney Int Rep 3:1239–1241. 10.1016/j.ekir.2018.08.002 30450445 10.1016/j.ekir.2018.08.002 PMC6224634

[13] Savige J, Storey H, Il Cheong H, Gyung Kang H, Park E, Hilbert P, Persikov A, Torres-Fernandez C, Ars E, Torra R, Hertz JM, Thomassen M, Shagam L, Wang D, Wang Y, Flinter F, Nagel M (2016) X-linked and autosomal recessive Alport syndrome: pathogenic variant features and further genotype-phenotype correlations. PLoS One 11:e0161802. 10.1371/journal.pone.0161802 27627812 10.1371/journal.pone.0161802 PMC5023110

[14] Savige J, Colville D, Rheault M, Gear S, Lennon R, Lagas S, Finlay M, Flinter F (2016) Alport syndrome in women and girls. Clin J Am Soc Nephrol 11:1713–1720. 10.2215/CJN.00580116 27287265 10.2215/CJN.00580116 PMC5012472

[15] Deltas C, Papagregoriou G, Louka SF, Malatras A, Flinter F, Gale DP, Gear S, Gross O, Hoefele J, Lennon R, Miner JH, Renieri A, Savige J, Turner AN (2023) Genetic modifiers of Mendelian monogenic collagen IV nephropathies in humans and mice. Genes 14:1686. 10.3390/genes14091686 37761826 10.3390/genes14091686 PMC10530214

[16] Mencarelli MA, Heidet L, Storey H, Geel M, Knebelmann B, Fallerini C, Miglietti N, Antonucci MF, Cetta F, Sayer JA, van den Wijngaard A, Yau S, Mari F, Bruttini M, Ariani F, Dahan K, Smeets B, Antignac C, Flinter F, Renieri A (2015) Evidence of digenic inheritance in Alport syndrome. J Med Genet 52:163–174. 10.1136/jmedgenet-2014-102822 25575550 10.1136/jmedgenet-2014-102822

[17] Savige J, Renieri A, Ars E, Daga S, Pinto AM, Rothe H, Gale DP, Aksenova M, Cerkauskaite A, Bielska O, Lipska-Zietkiewicz B, Gibson JT (2022) Digenic Alport syndrome. Clin J Am Soc Nephrol 17:1697–1706. 10.2215/CJN.03120322 35675912 10.2215/CJN.03120322 PMC9718039

[18] Zeng M, Di H, Liang J, Liu Z (2023) Effectiveness of renin–angiotensin–aldosterone system blockers in patients with Alport syndrome: a systematic review and meta-analysis. Nephrol Dial Transplant 38:2485–2493. 10.1093/ndt/gfad105 37218713 10.1093/ndt/gfad105

[19] Yamamura T, Horinouchi T, Nagano C, Omori T, Sakakibara N, Aoto Y, Ishiko S, Nakanishi K, Shima Y, Nagase H, Takeda H, Rossanti R, Ye MJ, Nozu Y, Ishimori S, Ninchoji T, Kaito H, Morisada N, Iijima K, Nozu K (2020) Genotype-phenotype correlations influence the response to angiotensin-targeting drugs in Japanese patients with male X-linked Alport syndrome. Kidney Int 98:1605–1614. 10.1016/j.kint.2020.06.038 32712167 10.1016/j.kint.2020.06.038

[20] Gross O, Licht C, Anders HJ, Hoppe B, Beck B, Tönshoff B, Höcker B, Wygoda S, Ehrich JHH, Pape L, Konrad M, Rascher W, Dötsch J, Müller-Wiefel DE, Hoyer P, Knebelmann B, Pirson Y, Grunfeld JP, Niaudet P, Cochat P, Heidet L, Lebbah S, Torra R, Friede T, Lange K, Müller GA, Weber M (2012) Early angiotensin-converting enzyme inhibition in Alport syndrome delays renal failure and improves life expectancy. Kidney Int 81:494–501. 10.1038/ki.2011.407 22166847 10.1038/ki.2011.407

[21] Gross O, Tönshoff B, Weber LT, Pape L, Latta K, Fehrenbach H, Lange-Sperandio B, Zappel H, Hoyer P, Staude H, König S, John U, Gellermann J, Hoppe B, Galiano M, Hoecker B, Ehren R, Lerch C, Kashtan CE, Harden M, Boeckhaus J, Friede T, German Pediatric Nephrology (GPN) Study Group and EARLY PRO-TECT Alport Investigators (2020) A multicenter, randomized, placebo-controlled, double-blind phase 3 trial with open-arm comparison indicates safety and efficacy of nephroprotective therapy with ramipril in children with Alport’s syndrome. Kidney Int 97:1275–1286. 10.1016/j.kint.2019.12.015 32299679 10.1016/j.kint.2019.12.015

[22] Osterberg L, Blaschke T (2005) Adherence to medication. N Engl J Med 353:487–497. 10.1056/NEJMra050100 16079372 10.1056/NEJMra050100

[23] Dew MA, Dabbs AD, Myaskovsky L, Shyu S, Shellmer DA, DiMartini AF, Steel J, Unruh M, Switzer GE, Shapiro R, Greenhouse JB (2009) Meta-analysis of medical regimen adherence outcomes in pediatric solid organ transplantation. Transplantation 88:736–746. 10.1097/TP.0b013e3181b2a0e0 19741474 10.1097/TP.0b013e3181b2a0e0 PMC2769559

[24] Dean AJ, Walters J, Hall A (2010) A systematic review of interventions to enhance medication adherence in children and adolescents with chronic illness. Arch Dis Child 95:717–723. 10.1136/adc.2009.175125 20522463 10.1136/adc.2009.175125

[25] McGrady ME, Hommel KA (2013) Medication adherence and health care utilization in pediatric chronic illness: a systematic review. Pediatrics 132:730–740. 10.1542/peds.2013-1451 23999953 10.1542/peds.2013-1451 PMC3784296

[26] Simoni JM, Asarnow JR, Munford PR, Koprowski CM, Belin TR, Salusky IB (1997) Psychological distress and treatment adherence among children on dialysis. Pediatr Nephrol 11:604–606. 10.1007/s004670050346 9323288 10.1007/s004670050346

[27] Sewitch MJ, Abrahamowicz M, Barkun A, Bitton A, Wild GE, Cohen A, Dobkin PL (2003) Patient nonadherence to medication in inflammatory bowel disease. Am J Gastroenterol 98:1535–1544. 10.1111/j.1572-0241.2003.07522.x 12873575 10.1111/j.1572-0241.2003.07522.x

[28] Blydt-Hansen TD, Pierce CB, Cai Y, Samsonov D, Massengill S, Moxey-Mims M, Warady BA, Furth SL (2014) Medication treatment complexity and adherence in children with CKD. Clin J Am Soc Nephrol 9:247–254. 10.2215/CJN.05750513 24262500 10.2215/CJN.05750513 PMC3913241

[29] Ibrahim N, Wong IC, Patey S, Tomlin S, Sinha MD, Jani Y (2013) Drug-related problem in children with chronic kidney disease. Pediatr Nephrol 28:25–31. 10.1007/s00467-012-2149-1 22451139 10.1007/s00467-012-2149-1

[30] Boeckhaus J, Hoefele J, Riedhammer KM, Tönshoff B, Ehren R, Pape L, Latta K, Fehrenbach H, Lange-Sperandio B, Kettwig M, Hoyer P, Staude H, Konrad M, John U, Gellermann J, Hoppe B, Galiano M, Gessner M, Pohl M, Bergmann C, Friede T, Gross O (2021) Precise variant interpretation, phenotype ascertainment, and genotype–phenotype correlation of children in the EARLY PRO-TECT Alport trial. Clin Genet 99:143–156. 10.1111/cge.13861 33040356 10.1111/cge.13861

[31] Boeckhaus J, Tönshoff B, Weber LT, Pape L, Latta K, Fehrenbach H, Lange-Sperandio B, Kettwig M, König S, John-Kroegel U, Gellermann J, Galiano M, Jami S, Pieper D, Dihazi GH, Hafke A, Kohl S, Liebau MC, König J, Haffner D, Gross O, Wallbach M (2025) Urinary Dickkopf-related protein 3 as a novel biomarker for kidney function decline in children with Alport syndrome. Pediatr Nephrol 40:2205–2213. 10.1007/s00467-025-06696-3 39904897 10.1007/s00467-025-06696-3 PMC12116715

[32] Boeckhaus J, Strenzke N, Storz C, Gross O (2020) Characterization of sensorineural hearing loss in children with Alport syndrome. Life (Basel) 10:360. 10.3390/life10120360 33352923 10.3390/life10120360 PMC7766141

[33] Boeckhaus J, Mohr L, Dihazi H, Tönshoff B, Weber LT, Pape L, Latta K, Fehrenbach H, Lange-Sperandio B, Kettwig M, Staude H, König S, John-Kroegel U, Gellermann J, Hoppe B, Galiano M, Haffner D, Rhode H, Gross O (2023) Ratio of urinary proteins to albumin excretion shifts substantially during progression of the podocytopathy Alport syndrome, and spot urine is a reliable method to detect these pathologic changes. Cells 12:1333. 10.3390/cells12091333 37174733 10.3390/cells12091333 PMC10177071

[34] Rhode H, Lüse A, Tautkus B, Nabity M, John-Kroegel U, Weigel F, Dost A, Schitke J, Metzing O, Böckhaus J, Rubel D, Kiess W, Gross O (2023) Urinary protein-biomarkers reliably indicate very early kidney damage in children with Alport syndrome independently of albuminuria and inflammation. Kidney Int Rep 8:2778–2793. 10.1016/j.ekir.2023.09.028 38106579 10.1016/j.ekir.2023.09.028 PMC10719601

[35] Gross O, Friede T, Hilgers R, Görlitz A, Gavénis K, Ahmed R, Dürr U (2012) Safety and efficacy of the ACE-inhibitor ramipril in Alport syndrome: the double-blind, randomized, placebo-controlled, multicenter phase III EARLY PRO-TECT Alport trial in pediatric patients. ISRN Pediatr 2012:436046. 10.5402/2012/436046 22811928 10.5402/2012/436046 PMC3395192

[36] Verho M, Luck C, Stelter WJ, Rangoonwala B, Bender N (1995) Pharmacokinetics, metabolism and biliary and urinary excretion of oral ramipril in man. Curr Med Res Opin 13:264–273. 10.1185/03007999509111551 7555035 10.1185/03007999509111551

[37] Meisel S, Shamiss A, Rosenthal T (1994) Clinical pharmacokinetics of ramipril. Clin Pharmacokinet 26:7–15. 10.2165/00003088-199426010-00002 8137599 10.2165/00003088-199426010-00002

[38] Akchurin OM, Schneider MF, Mulqueen L, Brooks ER, Langman CB, Greenbaum LA, Furth SL, Moxey-Mims M, Warady BA, Kaskel FJ, Skversky AL (2014) Medication adherence and growth in children with CKD. Clin J Am Soc Nephrol 9:1519–1525. 10.2215/CJN.01150114 24970873 10.2215/CJN.01150114 PMC4152804

[39] Weissberg-Benchell J, Glasgow AM, Tynan WD, Wirtz P, Turek J, Ward J (1995) Adolescent diabetes management and mismanagement. Diabetes Care 18:77–82. 10.2337/diacare.18.1.77 7698052 10.2337/diacare.18.1.77

[40] Krejci-Manwaring J, Tusa MG, Carroll C, Camacho F, Kaur M, Carr D, Fleischer AB, Balkrishnan R, Feldman SR (2007) Stealth monitoring of adherence to topical medication: adherence is very poor in children with atopic dermatitis. J Am Acad Dermatol 56:211–216. 10.1016/j.jaad.2006.05.073 17224366 10.1016/j.jaad.2006.05.073

[41] Röder E, Berger MY, De Groot H, Gerth van Wijk R (2008) Sublingual immunotherapy in youngsters: adherence in a randomized clinical trial. Clin Exp Allergy 38:1659–1667. 10.1111/j.1365-2222.2008.03060.x 18631346 10.1111/j.1365-2222.2008.03060.x

[42] Krishnan JA, Bender BG, Wamboldt FS, Szefler SJ, Adkinson NF, Zeiger RS, Wise RA, Bilderback AL, Rand CS (2012) Adherence to inhaled corticosteroids: an ancillary study of the Childhood Asthma Management Program clinical trial. J Allergy Clin Immunol 129:112–118. 10.1016/j.jaci.2011.10.030 22104610 10.1016/j.jaci.2011.10.030 PMC3350797

[43] Butz AM (2006) Evidence-based practice: what is the evidence for medication adherence in children? J Pediatr Health Care 20:338–341. 10.1016/j.pedhc.2006.05.003 16962441 10.1016/j.pedhc.2006.05.003

[44] Burgess E, Muirhead N, de Cotret PR, Chiu A, Pichette V, Tobe S, SMART (Supra Maximal Atacand Renal Trial) Investigators (2009) Supramaximal dose of candesartan in proteinuric renal disease. J Am Soc Nephrol 20:893–900. 10.1681/ASN.2008040416 10.1681/ASN.2008040416 PMC2663827 19211712

[45] Rovin BH, Barratt J, Heerspink HJL, Alpers CE, Bieler S, Chae DW, Diva UA, Floege J, Gesualdo L, Inrig JK, Kohan DE, Komers R, Kooienga LA, Lafayette R, Maes B, Małecki R, Mercer A, Noronha IL, Oh SW, Peh CA, Praga M, Preciado P, Radhakrishnan J, Rheault MN, Rote WE, Tang SCW, Tesar V, Trachtman H, Trimarchi H, Tumlin JA, Wong MG, Perkovic V et al (2023) Efficacy and safety of sparsentan versus irbesartan in patients with IgA nephropathy (PROTECT): 2-year results from a randomised, active-controlled, phase 3 trial. Lancet 402:2077–2090. 10.1016/S0140-6736(23)02302-4 37931634 10.1016/S0140-6736(23)02302-4

[46] Madison J, Wilhelm K, Meehan DT, Gratton MA, Vosik D, Samuelson G, Ott M, Fascianella J, Nelson N, Cosgrove D (2024) Ramipril therapy in integrin α1-null, autosomal recessive Alport mice triples lifespan: mechanistic clues from RNA-seq analysis. J Pathol 262:296–309. 10.1002/path.6231 38129319 10.1002/path.6231 PMC10872630

[47] Murphy AC, Proeschal A, Brightling CE, Wardlaw AJ, Pavord I, Bradding P, Green RH (2012) The relationship between clinical outcomes and medication adherence in difficult-to-control asthma. Thorax 67:751–753. 10.1136/thoraxjnl-2011-201096 22436168 10.1136/thoraxjnl-2011-201096

[48] Briesacher BA, Andrade SE, Fouayzi H, Chan KA (2008) Comparison of drug adherence rates among patients with seven different medical conditions. Pharmacotherapy 28:437–443. 10.1592/phco.28.4.43 18363527 10.1592/phco.28.4.437 PMC2737273

[49] Baumgartner PC, Haynes RB, Hersberger KE, Arnet I (2018) A systematic review of medication adherence thresholds dependent of clinical outcomes. Front Pharmacol 9:1290. 10.3389/fphar.2018.01290 30524276 10.3389/fphar.2018.01290 PMC6256123

[50] Gross O, Haffner D, Schaefer F, Weber LT (2024) SGLT2 inhibitors: approved for adults and cats but not for children with CKD. Nephrol Dial Transplant 39:907–909. 10.1093/ndt/gfae029 38308509 10.1093/ndt/gfae029

