# Supplementary material for: High adherence to angiotensin-converting enzyme inhibitor in children and adolescents with Alport syndrome: objective verification using liquid chromatography-mass spectrometry
Source: Pediatr Nephrol. 2025 Nov 22;41(4):1035–44. doi: 10.1007/s00467-025-07053-0 (PMC12953493; doi:10.1007/s00467-025-07053-0)
Supplement: Supplementary file 1 — (DOCX 35.2 KB) [file 467_2025_7053_MOESM1_ESM.docx]

Supplement

Table S1

| Pat. ID | Urin Sample | Verum/Placebo | Ramipril µg/L | Ramiprilat µg/L | Aggregation | Creatinine mg/dL |
| --- | --- | --- | --- | --- | --- | --- |
| 93_01_01 | 1 | V | 1,02 | 87,75 | 88,77 | 102 |
|  | 2 | V | 2,09 | 114,96 | 117,05 | >122 |
| 93_01_02 | 1 | V | 40,92 | 404,01 | 444,93 | 23,6 |
|  | 2 | V | 28,26 | 123,71 | 151,97 | 27,2 |
| 93_01_03 | 1 | V | 2,67 | 648,79 | 651,46 | 66,5 |
|  | 2 | V | 1,16 | 389,28 | 390,44 | 54,1 |
| 93_02_01 | 1 | V | 98,33 | 435,19 | 533,52 | 78,5 |
| 93_03_02 | 1 | V | <1 | 34,67 | 34,9 | 8,11 |
|  | 2 | V | <1 | 177,92 | 178,67 | 69,8 |
| 93_03_03 | 1 | P | <1 | <10 | <10 |  |
|  | 2 | V | 7,32 | 41,15 | 48,47 |  |
| 93_03_04 | 1 | V | 16,88 | 128,17 | 145,05 | 57,6 |
|  | 2 | V | 22,1 | 141 | 163,1 |  |
| 93_03_05 | 1 | P | <1 | <10 | <10 | 96,4 |
| 93_03_06 | 1 | V | 19,79 | 365,46 | 385,25 |  |
|  | 2 | V | 16,22 | 250,3 | 266,52 |  |
| 93_05_01 | 1 | V | 88,28 | 178,33 | 266,61 |  |
| 93_05_02 | 1 | V | 77,01 | 75,23 | 152,24 |  |
|  | 2 | V | <1 | <10 | <10* |  |
| 93_05_04 | 1 | V | 1,7 | 164 | 165,7 | 44 |
|  | 2 | V | 1,17 | 267 | 268,17 | 38,3 |
| 93_05_05 | 1 | V | 49,8 | 180 | 229,8 | 18,1 |
|  | 2 | V | <1 | 27,3 | 27,3* | 30,5 |
| 93_05_06 | 1 | V | <1 | 67,16 | 67,49 | 10,1 |
|  | 2 | V | <1 | 63,11 | 63,63 | 21,6 |
| 93_05_07 | 1 | V | 134,76 | 1378,2 | 1512,96 | 27,8 |
|  | 2 | V | 118,85 | 526,8 | 645,65 | 36,2 |
| 93_06_01 | 1 | V | 35,9 | 277,15 | 313,05 | 117 |
|  | 2 | V | <1 | 51,69 | 51,93 | 55,3 |
| 93_06_02 | 1 | V | <1 | <10 | <10* | 70,9 |
| 93_06_03 | 1 | V | 4,98 | 446,34 | 451,32 | 68,2 |
| 93_06_04 | 1 | V | <1 | 13,65 | 13,67* | 76,5 |
| 93_06_05 | 1 | P | <1 | <10 | <10 | 29,9 |
| 93_06_07 | 1 | V | <1 | 143,77 | 144,02 | 9,21 |
| 93_06_08 | 1 | V | 52,43 | 3252,63 | 3305,06 | >122 |
| 93_06_09 | 1 | V | 78,61 | 1157,86 | 1236,47 | 38,3 |
|  | 2 | V | 227,29 | 1877,29 | 2104,58 | 78,5 |
| 93_07_01 | 1 | V | <1 | 64,83 | 65,01 | 14,1 |
|  | 2 | V | <1 | 232,49 | 233,27 | 35,8 |
| 93_07_02 | 1 | V | <1 | 90,44 | 91,26 | 22,7 |
|  | 2 | V | 1,37 | 322,44 | 323,81 | 29,9 |
| 93_07_03 | 1 | V | 4,82 | 1443,3 | 1448,12 | 40,1 |
|  | 2 | V | 3,7 | 1075,96 | 1079,66 | 58,7 |
| 93_07_04 | 1 | V | 1,58 | 674 | 675,58 | 31,4 |
|  | 2 | V | 129 | 907 | 1036 | 41,6 |
| 93_07_05 | 1 | P | <1 | <10 | <10 | 28 |
|  | 2 | P | <1 | <10 | <10 | 78,4 |
| 93_07_06 | 1 | V | 154 | 2233 | 2387 | 93,5 |
|  | 2 | V | 278 | 938 | 1216 | 108 |
| 93_07_07 | 1 | V | 2,19 | 937,84 | 940,03 | 27,3 |
|  | 2 | V | 31,5 | 198,06 | 229,56 | 8,38 |
| 93_07_08 | 1 | V | 143 | 2401 | 2544 | 14,1 |
|  | 2 | V | 1,57 | 472 | 473,57 | 60,8 |
| 93_09_01 | 1 | P | <1 | <10 | <10 | 71 |
|  | 2 | V | 162 | 133 | 295 | 75 |
| 93_09_02 | 1 | P | <1 | <10 | <10 | 17,3 |
|  | 2 | P | <1 | <10 | <10 | 7,59 |
| 93_10_01 | 1 | V | 167 | 778 | 945 | 39,1 |
|  | 2 | V | 131 | 837 | 968 | 66,9 |
| 93_10_02 | 1 | P | <1 | <10 | <10 | 51,4 |
|  | 2 | P | <1 | <10 | <10 | 84,9 |
| 93_10_03 | 1 | V | 34,07 | 1306 | 1340,07 | 42,5 |
|  | 2 | V | 13,41 | 242 | 255,41 | 51,1 |
| 93_10_04 | 1 | V | 3,08 | 1338 | 1341,08 | 71,1 |
|  | 2 | V | 6,7 | 761 | 767,7 | 58 |
| 93_11_01 | 1 | V | <1 | 333 | 333,91 | 48,6 |
|  | 2 | V | 3,62 | 1235 | 1238,62 | 135 |
| 93_11_02 | 1 | V | 1,17 | 160 | 161,17 | 34,5 |
|  | 2 | V | 1,01 | 89,9 | 90,91 | 22,6 |
| 93_11_03 | 1 | V | <1 | 123,47 | 123,71 | 87,2 |
|  | 2 | V | 3,39 | 1390,63 | 1394,02 | 24,1 |
| 93_11_04 | 1 | V | 44,96 | 811,76 | 856,72 | 63,1 |
|  | 2 | V | <1 | 275,75 | 276,44 | 36,3 |
| 93_11_05 | 1 | V | 42,08 | 673,21 | 715,29 | 63,9 |
|  | 2 | V | 3,28 | 418,34 | 421,62 | 56,5 |
| 93_12_01 | 1 | V | 167,14 | 800,2 | 967,34 | 45,8 |
|  | 2 | V | 20,32 | 68,77 | 89,09 | 40,1 |
| 93_12_02 | 1 | P | <1 | <10 | <10 | 83,2 |
|  | 2 | V | 2,06 | 808,41 | 810,47 | 56,9 |
| 93_12_03 | 1 | V | 8,8 | 1676,63 | 1685,43 | 76,1 |
|  | 2 | V | 7,33 | 2402,33 | 2409,66 | 74,1 |
| 93_12_04 | 1 | V | 98,85 | 977,64 | 1076,49 | 15,5 |
|  | 2 | V | 165,39 | 1324,55 | 1489,94 | 25,2 |
| 93_12_05 | 1 | V | 4,98 | 1308,22 | 1313,2 | 82,3 |
|  | 2 | V | 1,05 | 399,65 | 400,7 | 73,7 |
| 93_13_01 | 1 | P | <1 | <10 | <10 | 35 |
|  | 2 | P | <1 | <10 | <10 | 90,8 |
| 93_13_02 | 1 | V | 2,57 | 399,62 | 402,19 | 77,2 |
|  | 2 | V | <1 | 112,77 | 113,35 | 56,7 |
| 93_13_04 | 1 | V | <1 | 92,04 | 92,45 | 35,3 |
|  | 2 | V | <1 | 212,4 | 213,39 | 88,7 |
| 93_14_02 | 1 | V | <1 | 662,68 | 663,63 | 57,5 |
|  | 2 | V | 1,03 | 937,62 | 938,65 | 63,4 |
| 93_14_03 | 1 | V | 87,22 | 909,6 | 996,82 | 129 |
|  | 2 | V | 64,63 | 291,47 | 356,1 | 63,5 |
| 93_14_04 | 1 | V | 1,59 | 918,68 | 920,27 | 87,3 |
|  | 2 | V | 78,51 | 1428,19 | 1506,7 | 68,8 |
| 93_15_01 | 1 | V | 1,19 | 693,82 | 695,01 | 130 |
|  | 2 | V | 6,03 | 608,08 | 614,11 | 23,9 |
| 93_15_03 | 1 | V | <1 | 167,5 | 167,98 | 125 |
|  | 2 | V | 1,71 | 229,72 | 231,43 | 35,8 |
| 93_16_01 | 1 | V | 7,46 | 362,66 | 370,12 | 32,1 |
|  | 2 | V | 5,56 | 496,79 | 502,35 | 37,1 |
| 93_16_02 | 1 | V | <1 | 71,13 | 71,39 | 23,5 |
| 93_16_03 | 1 | V | <1 | 712,91 | 713,71 | 93,5 |
|  | 2 | V | <1 | 44,41 | 44,52 | 23,1 |

S1: Results of Ramipril and Ramiprilat measurement in urine using an automated liquid chromatography-mass spectrometry method. *-marked values were considered non-adherent.

**Supplemental methods**

Determination of ramipril and ramiprilat in urine

*Chemicals and reagents*

Ramipril, Ramiprilat, D3-Ramipril (TRC, Canada), and D5-Ramiprilat (Alsachim, France) were used as standards. Calibration standards and quality control (QC) samples were prepared from stock solutions. Ramipril standards were 2.0, 20.0, and 200.0 µg/L; Ramiprilat standards were 20.0, 200.0, and 2000.0 µg/L. Ramipril QC samples were 5.0 and 50.0 µg/L; Ramiprilat QC samples were 50.0 and 500.0 µg/L. The internal standard solution, containing 40.0 µg/L Ramipril and 400.0 µg/L Ramiprilat, was prepared in 4% bovine albumin. Bovine albumin (Sigma Aldrich, Germany), acetonitrile, and methanol (Geyer, Germany), formic acid, and acetic acid (Fisher Chemical, Illkirch, France) were used. Ultrapure water was generated using a Sartorius purification system (Göttingen, Germany). All solutions were aliquoted and stored at -20°C.

*Sample preparation*

Samples were processed using a fully automated platform, from Shimadzu Corporation, composed of CLAM-2040 automation module coupled to a Nexera(TM)X2 UHPLC and LCMS-8060NX(TM) LC/MS/MS. HL-7 interface standards were used for bidirectional communication between the laboratory information system (LIS) (Dedalus, Germany) and the CLAM-LC/MS/MS (Shimadzu Corporation, Duisburg, Germany). Sample preparation, measurement, and quantification were performed automatically. 20 µL methanol, 20 µL urine, and 10 µL internal standard were dispensed onto a 0.45 µm PTFE filter, stirred (45 s, 1900 rpm), and protein precipitated with 60 µL acetonitrile. After stirring (45 s, 1900 rpm), the extract was filtered (-60 to -65 kPa, 45 s) and injected (1.0 µL) into the LC-MS/MS.

*LC-MS/MS conditions*

The chromatographic system consisted of two ShimadzuLC-30AD Pumps (NexeraX2), a CTO40AC oven and a SIL-40AC autosampler (Shimadzu Corporation, Duisburg, Deutschland). A Shim-pack Velox C18 column (2.7 µm, 50 × 2.1 mm) was used, with a gradient mobile phase of (A) water containing 0.02% acetic acid and 2 mM ammonium acetate, and (B) methanol containing 0.02% acetic acid and 2 mM ammonium acetate, at a flow rate of 500 µL/min. The gradient program was as follows: 0.00–0.3 min, 1% B; 0.3–0.7 min, 1–70% B; 0.7–1.35 min, 70% B; 1.4–2.0 min, 95% B; 2.1 min, 1% B. The column oven temperature was maintained at 60 °C. LC-MS/MS analysis was performed using an LCMS-8060 triple quadrupole mass spectrometer (Shimadzu Corporation, Duisburg, Germany) with positive electrospray ionization (ESI). The following interface parameters were used: interface voltage, 4 kV; nebulizing gas flow, 3 L/min; heating gas flow, 10 L/min; drying gas flow, 10 L/min; interface temperature, 300 °C; desolvation line (DL) temperature, 200 °C; heat block temperature, 400 °C; collision gas pressure, 270 kPa. Scheduled multiple reaction monitoring (MRM) was used for quantification, with a pause time of 1 ms and a dwell time of 5 ms.

*Validation results*

Calibration curves were linear (2.0–200.0 µg/L ramipril; 20.0–2000.0 µg/L ramiprilat). Calibration stability was assessed over 8 days with five independent calibration runs. The mean percentage deviation for calibrators and QC samples ranged from 0.6% to 6.51%. Within-run imprecision, expressed as the coefficient of variation (CV), was determined using QC samples. For ramipril, CVs were 0.87% at 5.0 µg/L and 1.53% at 50.0 µg/L. For ramiprilat, CVs were 7.2% at 50.0 µg/L and 1.15% at 500.0 µg/L. Between-run imprecision, also expressed as CV, was assessed over 8 days using the same QC concentrations. For ramipril, CVs were 1.92% and 2.25%. For ramiprilat, CVs were 3.54% and 2.12%. The lower limit of quantification (LLOQ) was determined to be 2.0 µg/L for ramipril (CV = 2.2%) and 20.0 µg/L for ramiprilat (CV = 3.43%).
