# Supplementary material for: High adherence to angiotensin-converting enzyme inhibitor in children and adolescents with Alport syndrome: objective verification using liquid chromatography-mass spectrometry
Source: Pediatr Nephrol. 2025 Nov 22;41(4):1035–44. doi: 10.1007/s00467-025-07053-0 (PMC12953493; doi:10.1007/s00467-025-07053-0)
Supplement: Supplementary file 2 — Graphical Abstract (PPTX 1.49 MB) [file 467_2025_7053_MOESM2_ESM.pptx]

## Slide 1
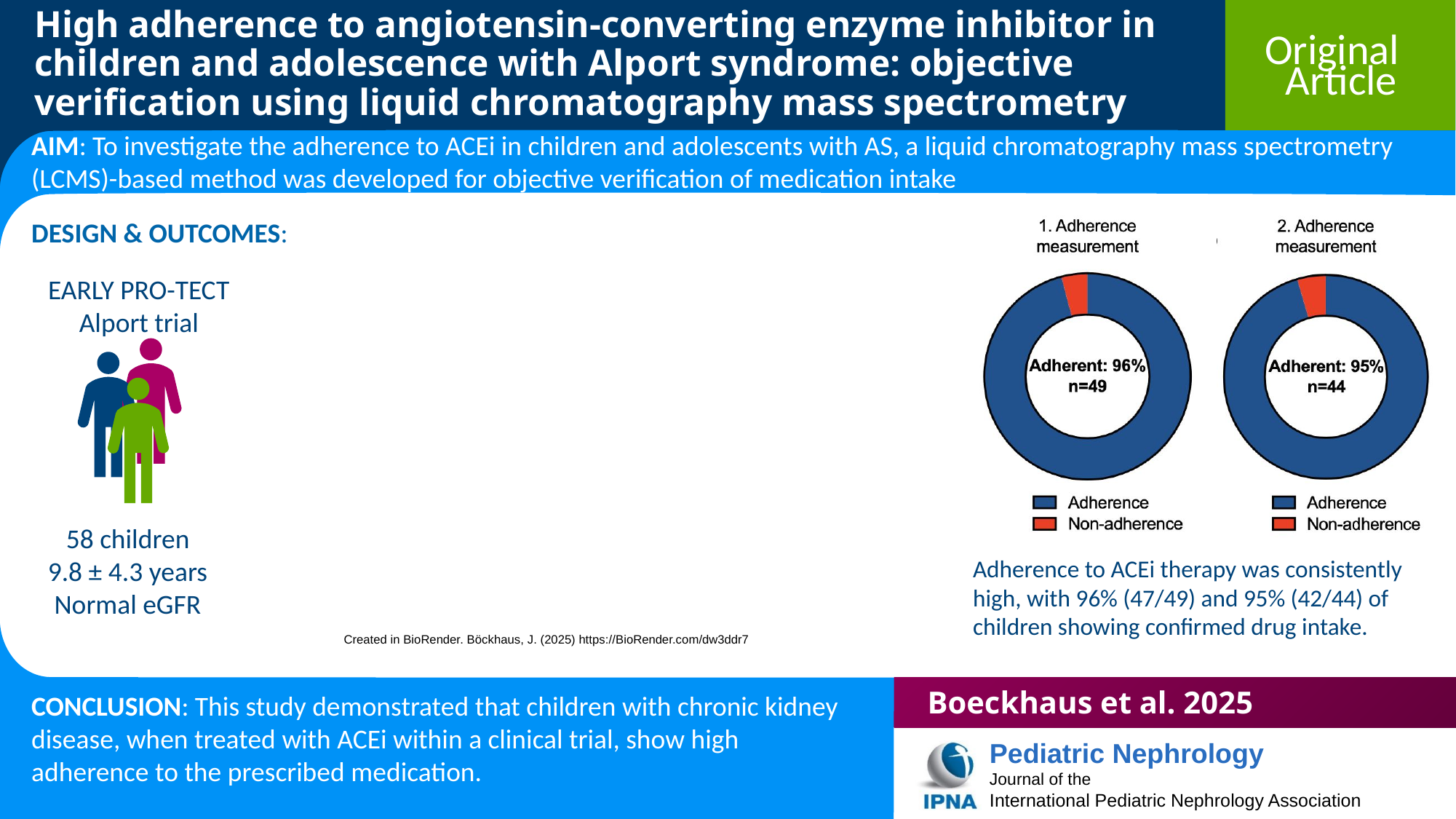

High adherence to angiotensin-converting enzyme inhibitor in children and adolescence with Alport syndrome: objective verification using liquid chromatography mass spectrometry
AIM: To investigate the adherence to ACEi in children and adolescents with AS, a liquid chromatography mass spectrometry (LCMS)-based method was developed for objective verification of medication intake
DESIGN & OUTCOMES:
EARLY PRO-TECT Alport trial
58 children
9.8 ± 4.3 years Normal eGFR
Adherence to ACEi therapy was consistently high, with 96% (47/49) and 95% (42/44) of children showing confirmed drug intake.
Created in BioRender. Böckhaus, J. (2025) https://BioRender.com/dw3ddr7
Boeckhaus et al. 2025
CONCLUSION: This study demonstrated that children with chronic kidney disease, when treated with ACEi within a clinical trial, show high adherence to the prescribed medication.
